# Circulating microRNAs in Atrial Fibrillation: Clinical Significance and Future Perspectives

**DOI:** 10.3390/medicina62061126

**Published:** 2026-06-09

**Authors:** Caglar Ozmen

**Affiliations:** Department of Cardiology, Faculty of Medicine, Çukurova University, 01330 Adana, Turkey; caglarozm@hotmail.com; Tel.: +90-532-583-9441

**Keywords:** atrial fibrillation, microRNA, circulating biomarkers, atrial fibrosis, diagnosis, prognosis, catheter ablation, stroke risk, precision medicine, epigenetics

## Abstract

Atrial fibrillation (AF) remains one of the most clinically demanding arrhythmias in contemporary cardiology—not because its mechanisms are unknown, but because what we know does not yet translate into precise, individualized management. Existing risk scores predict adverse outcomes reasonably well at the population level but perform inadequately for individual patients, and the molecular substrate driving disease progression remains largely invisible at the bedside. MicroRNAs (miRNAs), small non-coding RNA molecules of 20–25 nucleotides found stably in peripheral blood, have attracted considerable attention as potential biomarkers capable of bridging this gap. Their involvement in atrial fibrosis, electrical remodeling, and inflammatory signaling is mechanistically well-grounded. Whether this mechanistic plausibility can be translated into clinical utility is the central question this review addresses. We summarize the biological rationale for circulating miRNAs as AF biomarkers, review the most consistently replicated miRNA expression findings across clinical studies and meta-analyses, and appraise what the evidence supports—and what it does not—regarding diagnostic accuracy, prognostic value, and clinical decision-making applications. We also outline what the field needs to accomplish to move from promising findings to routine clinical use.

## 1. Introduction

The global burden of atrial fibrillation continues to grow. With prevalence estimates between 2% and 4% in adults—and projections suggesting more than a twofold increase over the coming decades as populations age and metabolic risk factors accumulate—AF has become a defining challenge of contemporary cardiovascular medicine [1,2]. The clinical consequences are well established: a fivefold increase in stroke risk, substantially elevated rates of heart failure and cognitive decline, and an independent contribution to mortality that persists after adjustment for most comorbidities [3,4].

Management frameworks have evolved considerably. The 2024 ESC guidelines and the 2023 ACC/AHA guidelines both reflect a shift toward earlier and more aggressive rhythm control, personalized anticoagulation, and integrated comorbidity management [5,6]. Yet despite this progress, certain fundamental clinical problems have proven stubbornly resistant to resolution. How can we reliably detect the atrial substrate before a stroke occurs? Which patients will develop recurrent arrhythmia after ablation, and which will not? And what does a CHA2DS2-VA score of 2 actually mean for the individual patient?

These questions share a common thread: they require information about the molecular state of the atrial myocardium—commonly referred to as the “atrial substrate”—that clinical variables alone cannot provide. The atrial substrate encompasses the structural, electrical, and inflammatory changes that accumulate within the atrial myocardium over time, including interstitial fibrosis, ion channel remodeling, and inflammatory activation. These changes create the electrophysiological environment in which AF initiates and perpetuates, yet they cannot be directly assessed by standard clinical tools such as the electrocardiogram or echocardiography. This is precisely the context into which circulating microRNA research has inserted itself over the past decade.

MicroRNAs are short, endogenous, non-coding RNA molecules that regulate gene expression post-transcriptionally [7,8]. Initially studied as intracellular regulators, they were subsequently found to circulate stably in plasma and serum—protected by exosomes, microvesicles, and protein complexes—making them amenable to minimally invasive measurement from peripheral blood [9,10]. Their stable detectability, combined with evidence that their expression profiles reflect pathological processes central to AF, made them appealing biomarker candidates almost immediately [11,12].

A substantial literature has now accumulated. Two meta-analyses published in 2023 provide the first quantitative synthesis of circulating miRNA findings in AF [13,14], and the first prospective studies linking miRNA pharmacology to AF management are underway [15]. At the same time, honest appraisal of the field reveals significant limitations: most studies are small, methodological heterogeneity is extreme, and no validated miRNA assay has been incorporated into any AF guideline. This review aims to present both the genuine promise and the honest shortcomings of circulating miRNAs in AF.

## 2. Clinical Challenges in Atrial Fibrillation Management

Before reviewing what miRNAs might offer, it is worth being explicit about what the clinical problems actually are. The case for a new biomarker must rest on a genuine unmet clinical need, not merely on the fact that something can be measured.

### 2.1. The Problem of Subclinical and Paroxysmal Atrial Fibrillation

A significant proportion of AF is asymptomatic. Subclinical AF refers to AF that is detected incidentally—typically by implantable cardiac monitors or wearable devices—in patients who have no AF-related symptoms and have not previously received an AF diagnosis. This entity carries intermediate thromboembolic risk, and the trials designed to determine whether anticoagulation is warranted have produced conflicting results. The NOAH-AFNET 6 trial found no net benefit from edoxaban in patients with device-detected atrial high-rate episodes, while the ARTESiA trial suggested possible benefit from apixaban in a similar population [16,17]. The difference in findings likely reflects differences in AF burden thresholds and outcome definitions, but the clinical bottom line is the same: better tools are needed to identify which subclinical AF patients harbor a genuinely high-risk atrial substrate, independent of how many hours their device recorded irregular activity [18,19].

A related diagnostic challenge concerns patients with documented paroxysmal AF—defined as AF confirmed on a 12-lead electrocardiogram for at least 10 s, or on a single-/multi-lead recording for at least 30 s—who have relatively infrequent, brief episodes and therefore spend most of their monitoring time in sinus rhythm. In such patients, a biomarker that reflects the structural state of the atrium—the degree of fibrosis, the extent of electrophysiological instability—could theoretically provide a continuous index of substrate severity even during sinus rhythm [20,21,22].

### 2.2. Recurrence After Catheter Ablation

Pulmonary vein isolation (PVI) remains the most effective rhythm control strategy for symptomatic AF, but its durability is far from guaranteed. Recurrence rates of 35–40% at 12 months in persistent AF are routinely reported [23], and the clinical scores designed to predict recurrence—APPLE, CAAP-AF, MB-LATER—perform only modestly, with area under the receiver operating characteristic curve (AUC) values typically in the 0.55–0.67 range [24,25]. These scores capture clinical risk factors but cannot assess what actually determines ablation success: the extent of atrial fibrosis and the reversibility of electrical remodeling [26]. Late gadolinium enhancement (LGE) magnetic resonance imaging (MRI) can assess fibrosis directly but requires specialized expertise and is unavailable in most ablation centers [27,28]. A circulating biomarker correlated with fibrosis burden would therefore address a genuine unmet need.

### 2.3. Residual Stroke Risk Under Anticoagulation

The CHA2DS2-VA score—the updated derivative recommended by the 2024 ESC guidelines—is a reasonable population-level tool but a limited individual-level one [5,29]. Even among patients receiving guideline-directed anticoagulation with direct oral anticoagulants (DOACs), major adverse cardiovascular events (MACE) occur at rates of 4–7% per year [30]. The inflammatory and thrombotic pathways driving this residual risk are not captured by age, hypertension status, or prior stroke history alone. Whether molecular biomarkers could provide incrementally useful information in this specific setting is one of the more clinically relevant questions in the field [31,32].

### 2.4. Monitoring Structural Progression over Time

AF is a progressive disease [33]. The transition from paroxysmal to persistent and ultimately permanent AF is not inevitable, but it is common, and it is driven by the accumulation of structural and electrical changes in the atrial myocardium that are largely invisible to routine clinical monitoring [34,35,36]. In summary, the four clinical problems outlined in this section—subclinical AF risk stratification, ablation outcome prediction, residual stroke risk stratification, and progressive substrate monitoring—represent genuine unmet needs that a validated circulating biomarker could, in principle, address.

## 3. Biological Basis of Circulating microRNAs in Atrial Fibrillation

### 3.1. Biogenesis and Stability in Circulation

The basic biology of miRNAs is well characterized. Transcribed from nuclear DNA as long primary transcripts (pri-miRNA), they undergo sequential processing by the Drosha/DGCR8 complex in the nucleus and the Dicer complex in the cytoplasm to yield mature molecules of 20–25 nucleotides. These are incorporated into the RNA-induced silencing complex (RISC) and directed toward complementary sequences in the 3′ untranslated regions of messenger RNAs [7,8,37]. The outcome is either target mRNA degradation or translational repression—a mechanism that gives individual miRNAs broad regulatory reach across multiple gene targets simultaneously.

**Figure 1:**
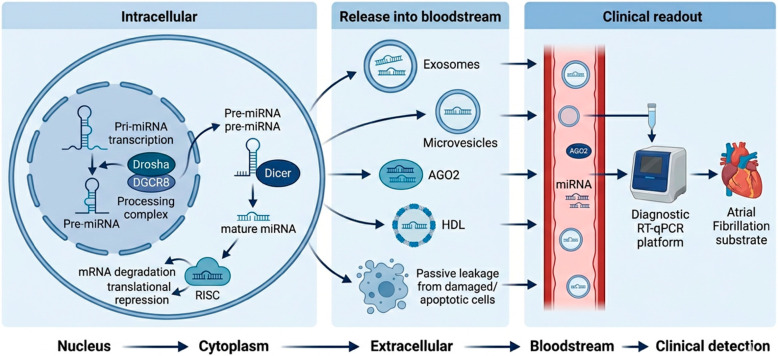
Biogenesis and release of circulating microRNAs: from cardiomyocyte to clinical biomarker. Intracellular miRNA processing (Drosha/DGCR8 → Dicer → RISC) and five extracellular release routes (exosomes, microvesicles, AGO2-complexes, HDL-associated, passive leakage from injured cells). Right panel: stability in peripheral blood, detection platforms, and clinical readout in AF. AGO2: argonaute-2 protein; AF: atrial fibrillation; APD: action potential duration; HDL: high-density lipoprotein; MACE: major adverse cardiovascular events; MVB: multivesicular body; NGS: next-generation sequencing; RISC: RNA-induced silencing complex; RT-qPCR: quantitative reverse transcriptase polymerase chain reaction; SMAD7: SMAD family member 7.

What makes circulating miRNAs analytically tractable is their stability. Released into the bloodstream through active secretion in exosomes and microvesicles, through passive leakage from damaged cells, or bound to argonaute-2 (AGO2) protein or high-density lipoprotein (HDL) particles, they resist ribonuclease degradation more effectively than most RNA types [9,10,38]. They remain stable at room temperature, survive repeated freeze–thaw cycles, and can be reliably detected from small plasma volumes using standard quantitative reverse transcriptase polymerase chain reaction (RT-qPCR) [39] [Figure 1]. That said, stability alone does not make something a useful biomarker. The more important questions are specificity and whether peripheral blood measurements faithfully represent cardiac tissue-level changes—both of which have complicated answers, as discussed below.

### 3.2. Technical Considerations

Circulating miRNA measurement from plasma or serum using RT-qPCR has become fairly standardized in research settings, with TaqMan-based assays being the most widely validated platform [40]. A persistent methodological challenge is normalization. Unlike mRNA measurement in tissue, plasma miRNA lacks universally validated endogenous controls. Most studies use synthetic spike-in controls—cel-miR-39 from Caenorhabditis elegans—added before RNA extraction to correct for isolation efficiency [41,42]. This approach is imperfect and introduces a source of inter-laboratory variability that has meaningfully complicated cross-study comparisons [43].

The sampling site is another underappreciated variable. Harada et al. [44] demonstrated, using simultaneous coronary sinus and femoral venous sampling, that miRNA profiles differ substantially between intracardiac and peripheral venous blood. A miRNA identified as elevated in peripheral plasma may therefore not reflect primarily cardiac pathology [44,45]. Future biomarker development efforts should account for this more systematically than most current studies have done.

### 3.3. Mechanistic Involvement in AF Pathophysiology

**Figure 2:**
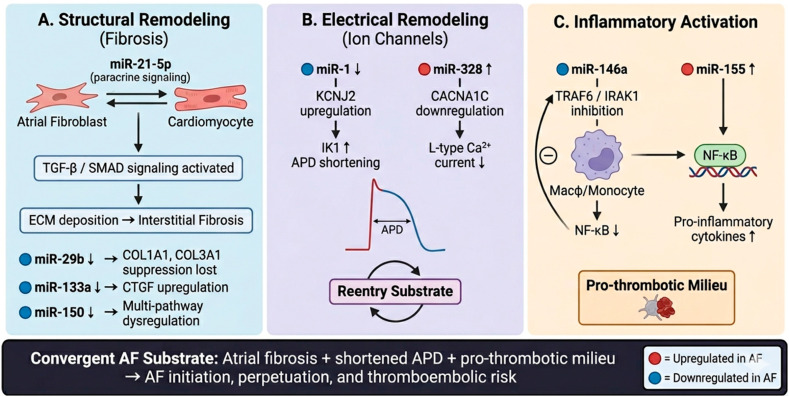
Mechanistic pathways of microRNAs in atrial fibrillation pathophysiology. Three interconnected remodeling processes and their key miRNA regulators. (**A**) Structural remodeling: pro-fibrotic miRNAs (miR-21, miR-146b-5p) promote TGF-β/SMAD signaling; anti-fibrotic miRNAs (miR-29b, miR-133a, miR-150) are downregulated in AF. (**B**) Electrical remodeling: downregulation of miR-1 and miR-133a leads to IK1 upregulation; miR-328 upregulation suppresses L-type Ca^2+^ current. (**C**) Inflammatory activation: miR-146a negatively regulates NF-κB signaling. Red tags: upregulated in AF; blue tags: downregulated in AF. AF: atrial fibrillation; APD: action potential duration; CACNA1C: L-type Ca^2+^ channel subunit alpha1 C; COL1A1/COL3A1: collagen types I/III; CTGF: connective tissue growth factor; Cx43: connexin-43; ECM: extracellular matrix; IK1: inward rectifier potassium current; KCNJ2: potassium inwardly rectifying channel J2; NF-κB: nuclear factor kappa-B; TGF-β: transforming growth factor-beta; TRAF6: TNF receptor-associated factor 6.

The case for miRNAs as AF biomarkers rests partly on their measurability but more fundamentally on their mechanistic involvement in the three processes that define the atrial substrate: atrial fibrosis, electrical remodeling, and inflammatory activation. Figure 2 summarizes these mechanistic pathways schematically.

#### 3.3.1. Atrial Fibrosis and Structural Remodeling

Interstitial fibrosis in the atrial myocardium creates the electrophysiological substrate for reentrant arrhythmias and is the structural feature most consistently associated with AF persistence and ablation failure [26,27]. The TGF-β/SMAD signaling axis is the central driver of this process, and miR-21 has been identified as a key modulator. Under tachyarrhythmic conditions, cardiomyocytes secrete miR-21-5p into the extracellular space, where it is taken up by neighboring fibroblasts and inhibits SMAD7—a negative regulator of TGF-β—thereby amplifying fibrotic gene expression in a paracrine loop [46,47]. miR-29b acts in the opposing direction, suppressing expression of collagens I and III and fibronectin-1; its downregulation in AF removes a brake on extracellular matrix accumulation [11,48]. miR-133a, another negative regulator of connective tissue growth factor (CTGF), is consistently reduced in AF tissue and has been identified as a candidate circulating biomarker in several cohort studies [13,14]. miR-146b-5p promotes fibrosis by repressing TIMP4, leading to upregulation of MMP9 and TGF-β [49]. miR-150, broadly reduced in AF, exerts multidirectional regulatory effects on fibrotic, inflammatory, and apoptotic pathways [50].

#### 3.3.2. Inflammatory Remodeling

Inflammation contributes to AF at multiple levels—initiation, maintenance, and the pro-thrombotic state that links AF to stroke [51,52]. miR-146a functions as a negative feedback regulator of NF-κB-driven inflammatory signaling through its targets TRAF6 and IRAK1. A functional single nucleotide polymorphism (SNP) in the miR-146a gene, rs2431697, modulates mature miR-146a levels and has demonstrated prognostic significance in AF independent of clinical risk scores [30]. miR-150 and miR-155 also participate in inflammatory pathway regulation in AF [50,53]. CRP, IL-6, and TNF-α—established inflammatory mediators in AF—share regulatory interconnections with several of these miRNAs, creating a feedback architecture that mirrors the clinical observation that AF and systemic inflammation co-evolve [54,55].

#### 3.3.3. Electrical Remodeling and Ion Channel Dysregulation

The progressive shortening of atrial action potential duration (APD) that characterizes AF-induced electrical remodeling involves dysregulation of multiple ion channels, and miRNAs contribute through direct targeting of channel-encoding genes [11,56]. miR-1, one of the most abundant cardiac miRNAs under normal conditions, is downregulated in persistent AF, leading to increased expression of KCNJ2 and consequently elevated inward rectifier potassium current (IK1), which abbreviates APD and facilitates reentry [57,58]. miR-328 modulates the L-type calcium channel alpha-1C subunit (CACNA1C) and is consistently upregulated in AF across multiple datasets [13,14,59]. miR-133a also affects connexin-43 and potassium channel-interacting protein expression, adding electrical remodeling to its already notable fibrosis-related roles [11,60].

## 4. Circulating microRNA Profiles in AF: Clinical Evidence and Significance

Table 1 provides a comprehensive overview of the key miRNA candidates discussed in this section. Table 2 summarizes the landmark prospective and observational clinical studies, including their designs, populations, and primary findings.

### 4.1. Meta-Analytic Findings

The two meta-analyses published in 2023 represent the best current synthesis of the clinical evidence and are worth examining carefully—not just for their conclusions, but for what their limitations reveal about the state of the field [13,14].

Menezes Junior et al. [13] analyzed six studies meeting inclusion criteria (plasma/serum samples, RT-qPCR quantification) and found an overall pooled odds ratio (OR) of 2.51 (95% CI 1.99–3.16) for miRNA dysregulation in AF. The most strongly associated individual miRNAs were miR-150 (OR 3.77), miR-20a-5p (OR 3.67), miR-133a (OR 2.77), hsa-miR-4443 (OR 2.32), miR-21 (OR 2.23), and miR-4798 (OR 1.90). The I^2^ statistic of 99% across studies indicates extreme heterogeneity, meaning that the pooled estimate should be interpreted with caution and should not be treated as a stable quantitative conclusion [64].

Rizal et al. [14], focusing specifically on diagnostic performance, identified five candidate biomarkers: miR-328, miR-223-3p, miR-21, miR-29b, and miR-1-5p. miR-328-3p showed the strongest single-biomarker diagnostic signal. miR-425-5p showed the highest reported sensitivity in individual cohort studies (sensitivity 0.96, diagnostic odds ratio 73.31) [14]. A separate systematic review examining miRNA profiles in cardioembolic stroke identified miR-21-5p as the most consistently upregulated miRNA across three independent studies [65].

### 4.2. Key Individual MicroRNA Candidates

#### 4.2.1. miR-21

miR-21 has been the most extensively studied circulating miRNA in AF, and the literature around it illustrates both the promise and the pitfalls of this research area. Galenko et al. found significantly elevated plasma miR-21 in AF patients on multivariate regression [13]. Hindricks et al. demonstrated that miR-21-5p levels correlate with bipolar voltage-map-derived fibrosis extent in AF patients undergoing ablation [46]. However, the miRhythm study found plasma miR-21 to be lower in AF patients compared to sinus rhythm controls, with levels increasing after successful ablation [61]. This directional inconsistency reflects genuine biological complexity across AF subtypes, sampling sites, and disease durations [66,67], and highlights the importance of carefully characterizing patient populations in future studies.

#### 4.2.2. miR-150

miR-150 shows more directional consistency across studies: it is reduced in AF relative to sinus rhythm in the majority of reports, and its post-ablation increase in the miRhythm study paralleled that of miR-21 [61]. Mechanistically, its downregulation affects at least 18 target genes across inflammatory, fibrotic, and apoptotic pathways [50], suggesting it functions as a broad homeostatic regulator whose loss reflects a general deterioration of atrial myocardial health. Its expression in intermediate monocytes also links it to systemic immune activation in AF [68].

#### 4.2.3. miR-328

miR-328-3p consistently emerges as the most diagnostically significant single miRNA in meta-analyses, with upregulation in AF across multiple independent cohorts and the strongest pooled diagnostic signal [14]. Its mechanistic rationale is directly tied to AF electrophysiology via CACNA1C targeting. A pooled OR of approximately 3.0 across multiple cohorts was confirmed by Huang et al. [69].

#### 4.2.4. miR-133a and miR-29b

These two miRNAs are worth considering together because they target overlapping fibrotic pathways from different angles. miR-133a suppresses CTGF and affects connexin-43 expression, contributing to both structural fibrosis and impaired intercellular coupling [70]. miR-29b inhibits extracellular matrix protein deposition by targeting collagen types I and III; its reduction reflects the failure of intrinsic anti-fibrotic mechanisms [11,48]. Their combined measurement may better capture the net fibrotic state of the atrium than either alone.

#### 4.2.5. miR-146a

miR-146a deserves special attention because it has the strongest prospective clinical validation of any miRNA in AF. The functional SNP rs2431697, which reduces mature miR-146a expression by approximately 50% in T-allele carriers, was shown in a prospective cohort of 901 anticoagulated AF patients to independently predict MACE when added to CHA2DS2-VASc and IL-6 [30]. This is arguably the most clinically credible miRNA finding in the AF literature to date.

### 4.3. The Cardiac Specificity Problem

One recurring limitation in this literature is the assumption that peripheral blood miRNA levels reflect cardiac pathology specifically. Harada et al. [44] addressed this directly using simultaneous intracardiac and peripheral venous sampling. Two novel candidates—miR-20b-5p and miR-330-3p—correlated with left atrial diameter in intracardiac samples but not in peripheral venous blood [44]. The cardiac specificity of peripheral blood miRNA measurements cannot be assumed and must be empirically established for each candidate.

### 4.4. Differential Expression Across AF Subtypes

Accumulating evidence suggests that miRNA expression profiles evolve with disease progression. miR-21 levels appear higher in persistent than paroxysmal AF, consistent with its role as an effector of fibrotic remodeling that accumulates with sustained arrhythmia [13]. The miRhythm study demonstrated differential plasma levels of miR-21 and miR-150 between paroxysmal and persistent AF [61]. These observations raise the prospect that miRNA profiles could serve not only as binary AF/no-AF classifiers, but as continuous markers reflecting the position along the paroxysmal-to-permanent disease continuum [71].

### 4.5. Diagnostic Potential—Promise and Realistic Limits

The diagnostic application of circulating miRNAs in AF encompasses two analytically distinct goals. The first is discriminating AF from sinus rhythm in symptomatic or screen-detected patients—a task for which existing methods already perform well, and where the incremental value of a blood test over a standard electrocardiogram is not immediately obvious [72]. The second goal, considerably more interesting clinically, is detecting atrial substrate abnormalities in patients who are currently in sinus rhythm, to identify those at high risk of future AF or at high risk of stroke despite no current arrhythmia documentation [22,73].

For the first goal, the meta-analytic evidence supports statistically significant associations but does not establish diagnostic accuracy adequate for clinical use. An OR of 2.5 is meaningful at the population level; it does not translate directly into an individual-level diagnostic test with acceptable sensitivity and specificity, particularly given the extreme inter-study heterogeneity [13,14,74]. For the second goal, there is promising but preliminary signal—particularly from studies correlating miR-21-5p with fibrosis burden [46] and from the pre-operative AF prediction work by Sharma et al. [23], which achieved an AUC of 0.83 using machine learning on pre-operative miRNA profiles in patients undergoing coronary artery bypass grafting (CABG)—but these remain proof-of-concept findings requiring larger, prospectively designed validation studies [62,75].

It is important to emphasize that the role of circulating miRNAs in AF diagnosis is not to replace or replicate electrocardiographic rhythm documentation. Rather, their potential diagnostic value lies in characterizing the atrial substrate and identifying at-risk patients before arrhythmia becomes clinically manifest or when intermittent monitoring is insufficient to capture it.

### 4.6. Prognostic Value

#### 4.6.1. Ablation Outcomes

The most mechanistically coherent and best-supported prognostic application is the use of pre-ablation miR-21-5p as a proxy for atrial fibrosis burden and a predictor of ablation success. Hindricks et al. [46] demonstrated in a prospective cohort of 175 patients that miR-21-5p levels correlated with bipolar voltage-map fibrosis extent and predicted freedom from AF recurrence at 12 months. The counterpoint comes from Lukas et al. [63], whose 90-patient prospective study failed to confirm pre-specified miRNA predictors of ablation success across the full cohort. These studies are not necessarily contradictory—they may reflect genuine heterogeneity in the miRNA–fibrosis–recurrence relationship across different patient populations and ablation approaches [76,77].

#### 4.6.2. Stroke and Major Adverse Cardiovascular Events

The prognostic data for MACE in anticoagulated AF patients center on miR-146a rs2431697. The prospective validation in 901 patients [30] is the most methodologically rigorous clinical evidence in this review—large enough to be meaningful, hypothesis-driven, biologically mechanistic, and validated in an already-treated population where residual risk is the clinical question [78]. miR-21′s role in cardioembolic stroke, supported by the Wang et al. systematic review [65], provides additional bioinformatic and mechanistic context. miR-106b, correlated with CHA2DS2 score in a 600-subject case–control study, demonstrated combined diagnostic and risk stratification utility [30,79].

## 5. Potential Role in Clinical Decision-Making

### 5.1. Anticoagulation Decisions

The most immediate clinical application for miRNA biomarkers in anticoagulation is augmenting the CHA2DS2-VA score for patients in the intermediate-risk zone where the benefit-to-bleeding-risk balance is most uncertain [5,29,80]. For patients with a CHA2DS2-VA score of 1, where the 2024 ESC guidelines offer a class IIa recommendation rather than a firm mandate [5], an independent molecular risk stratifier could support more individualized decisions. The rs2431697/miR-146a finding is the most relevant evidence here, but it requires replication in additional cohorts before it can inform clinical practice [30].

The miR-CRAFT study [15], which follows treatment-naïve AF patients initiating DOAC therapy to map miRNA expression changes longitudinally, represents a genuinely novel research direction: using miRNAs not to predict who needs anticoagulation, but to understand how anticoagulants modify the molecular substrate—and potentially to identify which patients derive the greatest benefit from specific agents [81]. For subclinical AF, where the evidence for anticoagulation remains contested following NOAH-AFNET 6 and ARTESiA [16,17], a circulating biomarker reflecting genuine atrial myopathy could help distinguish high-risk patients from those with incidental device-detected activity [82].

### 5.2. Patient Selection for Catheter Ablation

The practical case for pre-ablation miRNA measurement is straightforward in principle: if miR-21-5p correlates with LGE-MRI-derived fibrosis extent [46], and if fibrosis extent predicts ablation outcomes [26,27], then a blood test could serve as a non-invasive triage tool. Machine learning models incorporating miRNA inputs alongside imaging and clinical variables have demonstrated AUC values above 0.80 for ablation outcome prediction in exploratory analyses [24,83,84]. The critical next step is external validation of these models in independent multicenter datasets.

### 5.3. Disease Progression Monitoring

The miRhythm study’s observation that plasma miR-21 and miR-150 increase after successful ablation [61] suggests that these miRNAs reflect the functional state of the atrial substrate in a dynamic, treatment-responsive manner. If confirmed in larger studies with longer follow-up, serial miRNA measurement could become a component of post-ablation monitoring protocols [85,86]. In the context of rhythm versus rate control decision-making, miRNA profiles might help identify patients with ongoing atrial fibrotic progression who would benefit most from early, aggressive rhythm control [87].

### 5.4. Barriers to Clinical Implementation

A realistic assessment of the barriers to clinical miRNA use in AF must begin with the observation that the existing literature is largely composed of small, single-center, retrospective or cross-sectional studies with inconsistent findings. This represents the normal early stage of biomarker development, but it means the current evidence does not support clinical use [88,89]. Table 3 systematically summarizes the principal barriers and the methodological strategies proposed to address them.

## 6. Future Perspectives

### 6.1. The Validation Imperative

The single most important priority for the field is adequately powered prospective validation studies with pre-specified primary endpoints and standardized miRNA measurement protocols. The sample sizes required for stroke and MACE endpoints—several thousand patients with years of follow-up—exceed the reach of most individual research groups [88]. This argues strongly for embedding miRNA biobanking within existing large AF registries and outcome studies, rather than launching separate dedicated miRNA trials. The EORP-AF registry and similar European infrastructure would be natural candidates for this approach [90,91].

### 6.2. Multi-Biomarker Panel Strategies

**Figure 3:**
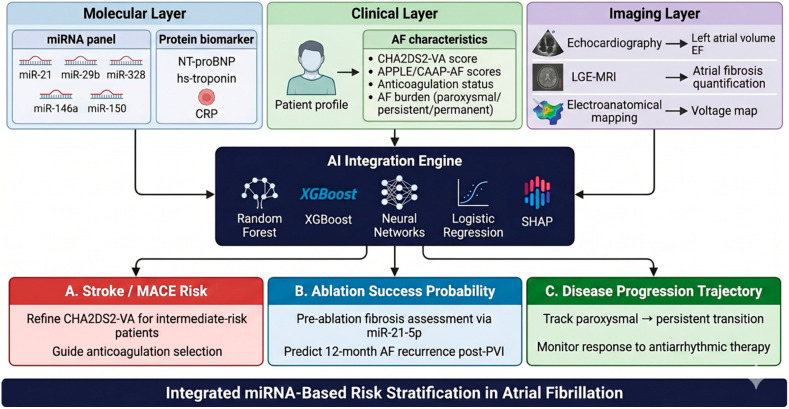
Proposed conceptual framework for integrated miRNA-based risk stratification in atrial fibrillation. Three input data layers (molecular: miRNA panel + protein biomarkers; clinical: patient profile + AF characteristics; Imaging: echocardiography + LGE-MRI + electroanatomical mapping) are integrated by AI-driven machine learning algorithms to generate individualized scores for three clinical decision domains: (**A**) stroke/MACE risk stratification; (**B**) ablation success probability; (**C**) disease progression trajectory. AF: atrial fibrillation; CHA2DS2-VA: stroke risk score (2024 ESC); LGE-MRI: late gadolinium enhancement MRI; LSTM: long short-term memory network; MACE: major adverse cardiovascular events; NT-proBNP: N-terminal pro-brain natriuretic peptide; PVI: pulmonary vein isolation; SHaAP: SHapley Additive exPlanations; SNP: single nucleotide polymorphism.

The evidence strongly suggests that no single miRNA will be sufficient as a standalone clinical AF biomarker [13,14,74]. A panel approach—combining structural remodeling markers (miR-21, miR-29b), electrical remodeling markers (miR-328, miR-1), inflammatory indicators (miR-146a, miR-150), and conventional protein biomarkers (NT-proBNP, hs-troponin, CRP)—is more likely to achieve the specificity and robustness needed for clinical translation [92,93]. Figure 3 illustrates a proposed conceptual framework for such an integrated panel approach.

### 6.3. Artificial Intelligence and Multi-Omic Integration

Machine learning approaches have already demonstrated that integrating multiple data types—clinical variables, imaging features, and biomarker levels—outperforms any single input for predicting AF-related outcomes [24,83,84,94]. Adding miRNA profiles to these multimodal models is a natural extension. The technical capacity exists; what is lacking is the prospective, harmonized, multi-center data needed to train and validate generalizable models [95]. Genome-wide multi-omic integration—linking circulating miRNA expression with DNA methylation, long non-coding RNA profiles, proteomics, and genomic variant data—is beginning to delineate the regulatory networks governing AF pathogenesis with unprecedented resolution [96,97]. The miR-CRAFT study [15] exemplifies this approach in the pharmacological context.

### 6.4. Therapeutic Applications

MiRNAs are not only biomarkers—they are also potential therapeutic targets. Antagomirs targeting miR-21 have attenuated fibrosis in animal models; miR-29b mimics have shown anti-fibrotic effects in experimental systems [11,98,99]. Whether these approaches can be translated to human cardiac disease without unacceptable off-target effects remains a significant challenge [100]. Tissue-specific delivery systems using adeno-associated viral vectors or lipid nanoparticle platforms are under development [101]. The intersection of diagnostic and therapeutic miRNA applications—using the same molecule as both a biomarker and a therapeutic target—is conceptually compelling, but the path to clinical implementation is long and the safety requirements are stringent [102].

## 7. Conclusions

The case for circulating miRNAs as clinically useful biomarkers in AF is mechanistically strong. The involvement of specific miRNAs—notably miR-21, miR-150, miR-328, miR-133a, miR-29b, miR-146a, and miR-1—in atrial fibrosis, electrical remodeling, and inflammatory signaling is grounded in solid experimental work, and their stable measurability in peripheral blood addresses a genuine need for non-invasive atrial substrate assessment. The meta-analytic evidence confirms statistically significant associations with AF that are unlikely to be artifactual [13,14].

The case for immediate clinical application, however, is weak. Methodological heterogeneity across existing studies is extreme, individual miRNA specificity is limited, and no miRNA measurement has been prospectively validated as improving clinical outcomes in AF patients. The strongest available evidence—the prospective validation of miR-146a rs2431697 as an independent MACE predictor [30] and the correlation of miR-21-5p with ablation-relevant fibrosis burden [46]—represents important progress but requires replication and expansion.

What the field needs now is not more discovery-phase studies identifying new candidate miRNAs, but rigorous, large-scale, prospective validation of the most promising candidates in well-characterized patient cohorts with hard clinical endpoints. That evidence will take years to generate and requires infrastructure and coordination beyond what individual research groups can provide. It is, however, the only path from mechanistic plausibility to clinical use—and it is the path that this field must now commit to.

## Figures and Tables

**Table 1:** Summary of key circulating microRNA candidates in atrial fibrillation: mechanistic roles, expression direction, and clinical significance.

miRNA	Direction in AF	Primary Mechanism	Key Target(s)	Diagnostic/Prognostic Role	Key Reference(s)
miR-21-5p	↑ tissue; variable in plasma	Atrial fibrosis (SMAD7/TGF-β, SPRY1/ERK)	SMAD7, SPRY1, PTEN	Fibrosis biomarker; post-ablation monitoring	[9,18,22]
miR-150	↓ plasma	Inflammation, apoptosis, fibrosis	Multiple (≥18 genes)	AF vs. sinus rhythm discrimination; post-ablation marker	[9,19,22]
miR-328-3p	↑ plasma	Electrical remodeling (APD shortening)	CACNA1C (L-type Ca^2+^ channel)	Strongest diagnostic signal in meta-analysis	[10,32]
miR-133a	↓ plasma and tissue	Fibrosis (CTGF), conduction (Cx43)	CTGF, KCNIP2, Cx43	Multi-panel component; structural marker	[8,9,10]
miR-29b	↓ plasma and tissue	Anti-fibrotic (ECM suppression)	COL1A1, COL3A1, FN1	Fibrotic burden indicator	[8,10]
miR-1-5p	↓ persistent AF	Electrical remodeling (IK1 upregulation)	KCNJ2 (Kir2.1)	APD shortening; diagnostic candidate	[8,10]
miR-146a	SNP-dependent (rs2431697)	NF-κB inflammatory signaling	TRAF6, IRAK1	Independent MACE predictor (n = 901)	[7]
miR-106b	↑ plasma and atrial tissue	Disease severity, thrombogenesis	MYL4	CHA2DS2 score correlation	[7]
miR-425-5p	↑ plasma	Ion channel regulation, Ca^2+^ signaling	Multiple	Highest sensitivity in single-study analysis (AUC 0.96)	[10]
miR-223-3p	↑ plasma	Inflammatory activation (neutrophil)	Multiple	Diagnostic candidate (meta-analysis)	[10]
miR-20b-5p	↑ intracardiac plasma	Structural remodeling (LA dilation)	Not fully defined	LA diameter correlation; cardiac-specific	[17]
miR-330-3p	↑ intracardiac plasma	Structural remodeling	Not fully defined	Novel candidate; intracardiac sampling	[17]

AF: atrial fibrillation; APD: action potential duration; CACNA1C: calcium voltage-gated channel subunit alpha1 C; COL1A1/COL3A1: collagen types I/III; CTGF: connective tissue growth factor; Cx43: connexin-43; ECM: extracellular matrix; FN1: fibronectin-1; IK1: inward rectifier potassium current; IRAK1: IL-1 receptor-associated kinase 1; KCNJ2/KCNIP2: potassium channel genes; LA: left atrium; MACE: major adverse cardiovascular events; MMP9: matrix metalloproteinase-9; MYL4: myosin light chain 4; POAF: post-operative atrial fibrillation; SMAD7: SMAD family member 7; SNP: single nucleotide polymorphism; SPRY1: sprouty homolog 1; SR: sinus rhythm; TGF-β: transforming growth factor-beta; TIMP4: tissue inhibitor of metalloproteinases 4; TRAF6: TNF receptor-associated factor 6; ↑: upregulated; ↓: downregulated.

**Table 2:** Summary of landmark clinical studies on circulating microRNAs in atrial fibrillation.

Study	Design	n	miRNA(s)	Sample	Key Finding	Clinical Endpoint
Hindricks et al., 2023 [18]	Prospective cohort (validation)	175	miR-21-5p	Peripheral arterial plasma	Correlation with bipolar voltage maps; predicts 12-month recurrence	AF recurrence after catheter ablation
McManus et al., 2015 [61] (miRhythm)	Prospective observational	47 (ablation); 31 (surgery)	miR-21, miR-150	Plasma	miR-21 and miR-150 twofold lower in AF; threefold increase post-ablation	Post-ablation biomarker dynamics
de los Reyes-García et al., 2023 [30]	Prospective cohort	901	miR-146a (rs2431697 SNP)	Plasma/Genomic	rs2431697 + CHA2DS2-VASc + IL-6 improves MACE prediction	MACE in anticoagulated AF patients
Harada et al., 2023 [44]	Prospective (intracardiac sampling)	24 (NGS); extended validation	miR-20b-5p, miR-330-3p	Coronary sinus + femoral vein plasma	Cardiac-specific miRNAs correlate with LA diameter; differ from peripheral levels	AF progression biomarkers
Sharma et al., 2025 [62]	Prospective (CABG)	15 (7 POAF/8 controls)	Panel of 10 (from 84 candidates)	Preoperative plasma	XGBoost AUC 0.83 for POAF prediction	Post-operative AF after CABG
Lukas et al., 2020 [63]	Prospective validation	90	Multiple (miR-125a, 10b, 601, 30a-3p, 199b)	Serum	Pre-specified miRNA predictors not confirmed; subgroup signal only	AF recurrence 12 months post-ablation
Ragia et al., 2024 [15] (miR-CRAFT)	Prospective longitudinal	Ongoing	Multiple (+DNA methylation)	Plasma (serial)	miRNA/methylation changes with DOAC initiation; epigenetic pharmacology	DOAC response in naïve AF patients

AF: atrial fibrillation; AUC: area under the receiver operating characteristic curve; CABG: coronary artery bypass grafting; DOAC: direct oral anticoagulant; LA: left atrium; MACE: major adverse cardiovascular events; NGS: next-generation sequencing; OR: odds ratio; POAF: post-operative atrial fibrillation; RT-qPCR: quantitative reverse transcriptase polymerase chain reaction; SNP: single nucleotide polymorphism; SR: sinus rhythm.

**Table 3:** Principal barriers to clinical implementation of circulating microRNA biomarkers in atrial fibrillation and proposed solutions.

Barrier	Current Status	Proposed Solution
Methodological heterogeneity (I^2^ = 99%)	Studies use different platforms, normalization methods, and sampling protocols	Harmonized pre-analytical standard operating procedures; multi-center prospective design
Limited disease specificity of single miRNAs	miR-21 and miR-150 are dysregulated in cancer, heart failure, and diabetes mellitus	Multi-miRNA panels with mechanistic complementarity; confounder adjustment
No validated normalization standard	cel-miR-39 spike-in is most widely used but not universally endorsed	International consortium for plasma miRNA reference standards
Sampling site confounds cardiac specificity	Peripheral venous blood dilutes cardiac-specific signal	Exosome surface profiling; cardiac-specific miRNA combinations
Small study sizes; single-center designs	Most studies include fewer than 200 participants; independent replication is rare	Embed miRNA biobanking in large AF registries (e.g., EORP-AF)
No outcome-driven prospective validation	No randomized controlled trial demonstrating that miRNA-guided decisions improve outcomes	Large prospective registry-embedded studies with hard clinical endpoints
Laboratory infrastructure not clinical-grade	RT-qPCR requires specialized equipment and expertise	Point-of-care microfluidic biosensor platforms under development

DOAC: direct oral anticoagulant; DM: diabetes mellitus; EORP-AF: EURObservational Research Programme Atrial Fibrillation; HF: heart failure; RCT: randomized controlled trial; RT-qPCR: quantitative reverse transcriptase polymerase chain reaction; SOP: standard operating procedure.

## Data Availability

No new data were created or analyzed in this study. Data sharing is not applicable to this article.

## Abbreviations

The following abbreviations are used in this manuscript:

ACC	American College of Cardiology
AGO2	Argonaute-2 protein
AF	Atrial fibrillation
AHA	American Heart Association
APPLE	Age, Prior stroke/TIA, Persistent AF, Early recurrence, Left atrial diameter score
APD	Action potential duration
AUC	Area under the receiver operating characteristic curve
CABG	Coronary artery bypass grafting
CACNA1C	Calcium voltage-gated channel subunit alpha1 C
CHA2DS2-VA	Stroke risk score (2024 ESC guidelines)
COL1A1	Collagen type I alpha 1
COL3A1	Collagen type III alpha 1
CRP	C-reactive protein
CT	Computed tomography
CTGF	Connective tissue growth factor
Cx43	Connexin-43
DECAAF	Delayed Enhancement MRI Determinant of Successful Radiofrequency Catheter Ablation of Atrial Fibrillation
DGCR8	DiGeorge syndrome critical region gene 8
DM	Diabetes mellitus
DOAC	Direct oral anticoagulant
ECM	Extracellular matrix
EORP-AF	EURObservational Research Programme Atrial Fibrillation
ERK	Extracellular signal-regulated kinase
ESC	European Society of Cardiology
FN1	Fibronectin-1
HAS-BLED	Hypertension, Abnormal renal/liver function, Stroke, Bleeding history, Labile INR, Elderly, Drugs/alcohol score
HDL	High-density lipoprotein
HRS	Heart Rhythm Society
hs-CRP	High-sensitivity C-reactive protein
IK1	Inward rectifier potassium current
IL-6	Interleukin-6
IRAK1	Interleukin-1 receptor-associated kinase 1
KCNIP2	Potassium channel interacting protein 2
KCNJ2	Potassium inwardly rectifying channel subfamily J member 2
LA	Left atrium
LGE-MRI	Late gadolinium enhancement magnetic resonance imaging
LSTM	Long short-term memory network
MACE	Major adverse cardiovascular events
MAP kinase	Mitogen-activated protein kinase
miRNA/miR	MicroRNA
MMP9	Matrix metalloproteinase-9
MVB	Multivesicular body
NF-κB	Nuclear factor kappa-light-chain-enhancer of activated B cells
NGS	Next-generation sequencing
NT-proBNP	N-terminal pro-brain natriuretic peptide
OR	Odds ratio
POAF	Post-operative atrial fibrillation
PVI	Pulmonary vein isolation
RCT	Randomized controlled trial
RISC	RNA-induced silencing complex
RNase	Ribonuclease
RT-qPCR	Quantitative reverse transcriptase polymerase chain reaction
SCAF	Subclinical atrial fibrillation
SHAP	SHapley Additive exPlanations
SMAD7	SMAD family member 7
SNP	Single nucleotide polymorphism
SOP	Standard operating procedure
SPRY1	Sprouty homolog 1
TGF-β	Transforming growth factor-beta
TIMP4	Tissue inhibitor of metalloproteinases 4
TNF-α	Tumor necrosis factor-alpha
TRAF6	TNF receptor-associated factor 6
